# Drug Transporters and Metabolizing Enzymes in Antimicrobial Drug Pharmacokinetics: Mechanisms, Drug–Drug Interactions, and Clinical Implications

**DOI:** 10.3390/biom15060864

**Published:** 2025-06-13

**Authors:** Kaili Lin, Ruoqing Wang, Tong Li, Yawen Zuo, Shilei Yang, Deshi Dong, Yanna Zhu

**Affiliations:** Department of Pharmacy, First Affiliated Hospital of Dalian Medical University, Dalian 116011, China; 13963751074@163.com (K.L.); wakahare_w@163.com (R.W.); ltong20012024@163.com (T.L.); zyw000602@163.com (Y.Z.); yangshilei@dmu.edu.cn (S.Y.); dongdeshi@dmu.edu.cn (D.D.)

**Keywords:** antimicrobial drugs, drug transporters, metabolizing enzymes, pharmacokinetics, drug–drug interactions

## Abstract

Drug transporters and metabolizing enzymes are integral components of drug disposition, governing the absorption, distribution, metabolism, and excretion (ADME) of pharmaceuticals. Their activities critically determine therapeutic efficacy and toxicity profiles, particularly for antimicrobial agents, one of the most widely prescribed drug classes frequently co-administered with other medications. Emerging evidence highlights the clinical significance of the drug–drug interactions (DDIs) mediated by these systems, which may alter antimicrobial pharmacokinetics, compromise treatment outcomes, or precipitate adverse events. With the continuous introduction of novel antimicrobial agents into clinical practice, the role of drug transporters and metabolizing enzymes in the pharmacokinetics of antibiotics and the DDIs between antibiotics and other drugs mediated by these transporters and enzymes are important to determine in order to provide a theoretical basis for the safe and effective use of antimicrobial drugs in clinical use.

## 1. Introduction

**Figure 1:**
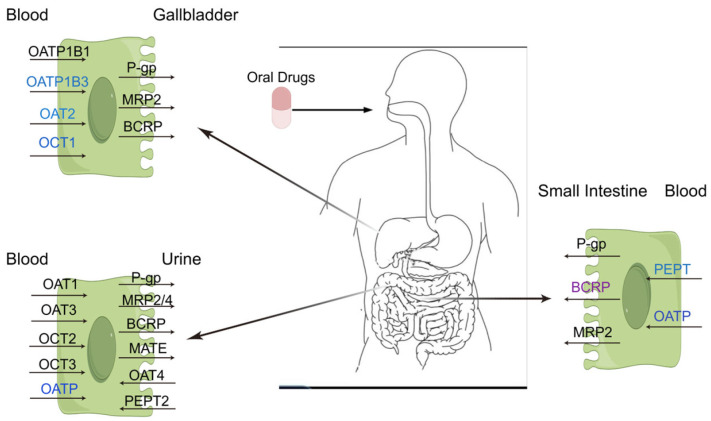
Major drug transporters in the body. OAT1—organic anion transporter 1; OAT2—organic anion transporter 2; OAT3—organic anion transporter 3; OATP1B1—organic anion transporter polypeptide 1B1; OATP1B3—organic anion transporter polypeptide 1B3; OCT1—organic cation transporter 1; OCT2—organic cation transporter 2; OCT3—organic cation transporter 3; PEPT2—peptide transporter 2; MATE—multidrug and toxin extrusion protein; MRP2—multidrug resistance-associated protein 2; MRP4—multidrug resistance-associated protein 4; P-gp—P-glycoprotein; BCRP—breast cancer resistance protein. Efflux transporters/carriers are highlighted in purple, and influx carriers are highlighted in blue.

One of the main research areas of pharmacokinetics is the body’s handling of drugs and the processing of drugs in the body, including their absorption, distribution, metabolism, and excretion [1]. Drug absorption refers to the entry of drugs into the bloodstream from the administration site [2], which is influenced by the drug’s physicochemical properties, route of administration, and blood flow at the absorption site. The process by which the drug is absorbed and subsequently circulates to various tissues and organs is termed distribution [1]. This distribution is affected by plasma protein binding, tissue affinity, physiological barriers, and transporter activity. The term metabolism describes the chemical alterations of the drug’s structure after it reaches various tissues and organs [3]. The primary metabolic sites include the liver, small intestine, kidney, lung, and brain, with the liver being the most critical organ [4]. Cytochrome P450 (CYP450) enzymes form a superfamily found primarily in the liver and other organs such as the intestines, kidneys, lungs, and brain [5]. CYPs mediate the oxidative biotransformation of 70–80% of marketed drugs [6]. Excretion refers to the elimination of drugs and their metabolites from the body [7]. Drug transporters are transmembrane proteins widely distributed in organs such as the intestinal tract, liver, and kidneys and play a critical role in vivo drug processes [8]. Drug transporters can be classified into two major families based on transport mechanisms: the soluble carrier (SLC) family, which includes secondary active transporters, and the ATP-binding cassette (ABC) transporters, which are primary active transporters [1,8,9]. Additionally, drug transporters can also be divided into uptake transporters and efflux transporters according to their transport directions and energy requirements. Uptake transporters primarily facilitate drug entry into cells, thereby increasing intracellular substrate concentrations [10]. Uptake transporters include the organic anion transporting polypeptide (OATP) [11], organic anion transporter (OAT) [12], organic cation transporter (OCT) [13], and oligopeptide transporter (PEPT) [14]. Efflux transporters, on the other hand, utilize energy from adenosine triphosphate (ATP) hydrolysis to expel substrates out of cells, reducing the intracellular substrate levels. Major efflux transporters include P-glycoprotein (P-gp) [15], multidrug resistance-associated protein (MRP) [16], multidrug and toxin extrusion protein (MATE) [17], and breast cancer resistance protein (BCRP) [18]. The distribution of the main drug transporters in the human body is shown in Figure 1.

Antibacterial agents refer to a class of drugs used to treat pathogenic infections [19], mainly including antibiotics, synthetic antibacterial drugs, antifungals, antivirals, and antiparasitic drugs [20]. Among these, antibiotics are the most widely used clinically, with a prescription rate significantly higher than other drug classes. Due to the extensive use of antibiotics and the rising prevalence of microbial resistance, new antibacterial agents are continually being introduced into clinical practice [21]. Novel antibiotics developed in recent years include cephalosporins (e.g., ceftobiprole [22]), β-lactam/β-lactamase inhibitor combination (e.g., ceftazidime–avibactam [23], meropenem–vaborbactam [24], sulbactam–durlobactam [25]), next-generation fluoroquinolones (e.g., nemonoxacin [26], sitafloxacin [27]), and the glycylcycline eravacycline [28], all demonstrating proven efficacy against resistant pathogens. The pharmacokinetics of most antibiotics are influenced by drug transporters and drug-metabolizing enzymes (Table 1, Table 2 and Table 3). Fungi cause a variety of diseases in humans, including allergic syndromes, superficial infections, disfiguring conditions, and life-threatening invasive fungal diseases, affecting over 1 billion people worldwide. Currently, the main classes of antifungal drugs are polyenes, azoles, echinocandins, and the pyrimidine analogue 5-fluorocytosine [29]. The high prevalence of viral epidemics has driven advancements in antiviral drug research. Antiviral agents can be primarily categorized into the following types: neutralizing antibodies, neutralizing recombinant soluble human receptors, antiviral CRISPR/Cas systems, interferons, antiviral peptides, antiviral nucleic acid polymers, and antiviral small molecules [30]. Furthermore, as antibacterial agents are frequently co-administered with other medications, clinically significant DDIs may arise. DDIs are defined as alterations in the pharmacokinetics, pharmacodynamics, or toxicity of a drug caused by the concomitant use of two or more medications. These interactions are a critical concern in clinical practice, particularly for patients receiving polypharmacy (the simultaneous use of multiple drugs). DDIs may lead to outcomes ranging from reduced therapeutic efficacy to severe adverse events [31,32]. Therefore, this review aims to summarize the relationships between antibacterial agents, drug transporters, and drug-metabolizing enzymes. Understanding DDIs mediated by these transporters and enzymes is essential for optimizing the safe and effective clinical use of antibacterial agents.

## 2. Uptake Transporters with Antibacterial Agents

### 2.1. Organic Anion Transporter (OAT)

OATs take up organic anions through an exchange mechanism that couples the influx of organic anions with the efflux of intracellular dicarboxylates [12]. The OATs primarily include hOAT1, hOAT2, hOAT3, and hOAT4. Among these, hOAT1, hOAT2, and hOAT3 are localized to the basolateral membrane of the proximal tubule, whereas hOAT4 is expressed on the apical brush border membrane. These transporters collectively mediate both the uptake and efflux of tetracycline [33]. Studies have demonstrated that tetracycline can inhibit organic anion uptake by OAT1, OAT2, and OAT4; oxytetracycline, minocycline, and doxycycline can inhibit organic anion uptake by OAT1, whereas oxytetracycline and minocycline but not doxycycline can inhibit organic anion uptake by OAT2 [33]. OATs are also involved in the renal excretion process of β-lactam antibiotics. For example, studies using OAT1-transfected Xenopus oocytes found that cefazolin, cefotiam, and cephalexin were substrates for OAT1 [33,34]. Cefotiam and cefdinir were identified as OAT3 substrates in OAT3-HEK293 cells [35]. Apart from OAT1 and OAT3, OAT2 also has the capacity to accommodate various organic anions and transport numerous bioactive agents [36]. There are many antibiotics among the well-characterized drug substrates of OAT2, such as cefotaxime, erythromycin, and tetracycline [36]. In the new β-lactam/β-lactamase inhibitor combination meropenem–vaborbactam, meropenem serves as a substrate for OAT1 and OAT3 in the renal proximal tubule [37]. The advanced fluoroquinolone nemonoxacin can also serve as a substrate for OAT1 and OAT3 [38].

Due to the broad substrate specificity of OATs, OAT-mediated DDIs occur when two OAT substrates are co-administered. This risk is particularly significant for drugs reliant on OAT1 or OAT3 for renal elimination [39]. Combining these drugs with furosemide and other diuretics reduces OAT-mediated proximal tubule secretion, thereby impairing their efficacy [40]. Emerging DDIs are not universally deleterious: for example, methylsulfonate reduces penicillin’s renal excretion via OAT inhibition, elevating systemic exposure to the antibiotic [39]. Studies have shown that after absorption, penicillin mainly distributes in the plasma and has difficulty passing through the blood–brain barrier. It is mainly excreted rapidly through the kidneys [41]. When deep brain function is impaired, excretion is significantly delayed. OAT3 is the main transporter responsible for penicillin excretion, so the interaction between methylsulfonate and penicillin primarily occurs via OAT3 [40]. Studies have also shown that puerarin enhances methotrexate exposure by inhibiting OAT1 and OAT3, thereby improving its bioavailability without increasing renal toxicity [42]. The nephrotoxicity of adefovir and cidofovir is related to the uptake mediated by OAT1. When using cidofovir clinically, it is necessary to combine it with the OAT inhibitor ciprofloxacin to reduce the nephrotoxicity [43].

### 2.2. Organic Anion Transporting Polypeptide (OATP)

OATP is widely distributed in various organs, including the liver, intestine, and blood–brain barrier. The liver primarily expresses OATP1A2, OATP1B1, OATP1B3, and OATP2B1. Among these, OATP1B1 and OATP1B3 play a crucial role in hepatic drug elimination, thereby altering drug pharmacokinetics [44]. For example, statins, lipid-lowering drugs, and cephalosporin antibiotics are substrates and are transported by these transporters [45,46].

OATPs also play an important role in the uptake and distribution of quinolones. OATP1A2 is expressed in Xenopus oocytes, and experiments have shown that ciprofloxacin and levofloxacin are transported by this transporter, confirming them as OATP1A2 substrates [47]. Additionally, studies in rats demonstrated that Oatp1a5 mediates the intestinal uptake of ciprofloxacin, while naringin inhibits the uptake of ciprofloxacin in Xenopus oocytes expressing Oatp1a5, with an IC50 of 18 µM [48]. Rifampicin serves as an inhibitor of OATP1B1 [49] and OATP1B3 [50]. When rifampicin is combined with erythromycin and clarithromycin, their uptake via OATP1B1 and OATP1B3 decreases by 65% and 45%, respectively [51]. However, rifampicin does not reduce the total blood concentrations of these macrolides. Therefore, to determine whether OATPs/Oatps play a significant role in macrolide transport, researchers have conducted further investigations. Several studies have demonstrated that macrolides act as inhibitors of OATPs/Oatps. For example, in OATP1B1-HEK293 cells and OATP1B3-HEK293 cells, all macrolides (except azithromycin) inhibit the uptake of typical substrates such as bilirubin, sulfobromophthalein (BSP), and pravastatin in a concentration-dependent manner [52]. After entering the human body, azithromycin mainly accumulates in phagocytes. It is widely distributed in tissues and has a rapid clearance rate [53]. In addition, Garver et al. [51] demonstrated that azithromycin and clarithromycin significantly inhibit taurocholate uptake in Oatp1a5-MDCK transfected cells. Similar results were also confirmed in another study: azithromycin and clindamycin strongly inhibited the uptake of taurocholate sodium via Oatp1a5, with Ki values of 3.3 µM and 2.4 µM, respectively. However, azithromycin and clarithromycin did not significantly inhibit OATP2B1 transport of estrone 3-sulfate (a typical substrate of OATP). Furthermore, these macrolides were not transported by OATP2B1/Oatp2b1 transfected cells [52]. Therefore, the effects of macrolides vary across different OATP/Oatp transporters.

Most β-lactam antibiotics are transported into the liver via carrier-mediated mechanisms. Studies have shown that the uptake process of nafcillin in rat liver cells is concentration-dependent and saturable, with a Km value of 210 μM. Further verification was conducted using the Xenopus oocyte model. Nafcillin can be transported by multiple Oatp subtypes. Among them, Oatp2 (Oatp1a4) has the highest affinity (Km = 198 μM), which is significantly superior to Oatp1 (Km = 4120 μM) and Oatp4 (Km = 1570 μM). However, no transport activity was observed in hOAT2. The analysis of relative activity factors confirmed that OATP2 plays a dominant role [54]. In addition, six beta-lactam drugs including cefradine, cefoperazole, cefazolin, cefsulodin, cephalexin, and cefmetazole have also been confirmed as substrates of Oatp2, but cefotaxime and ceftriaxone did not show transport dependence [54]. Another study used Xenopus oocytes to transfect human OATPs cells and found that nafcillin had a Km value of 74 μM and 11 μM for OATP1B1 and OATP1B3, respectively. There is also a main role for OATP1B3, with 20.5% and 53.3% contribution rates to nafcillin uptake in the human liver. They tested whether β-lactam antibiotics can be transported by OATP1B3; OATP1B1 can transport cefazolin and cefoperazone but not cefmetazole, cefradine, and cephalexin. OATP1A4 in rats and OATP1B3 in humans have similar roles in nafcillin uptake in the liver [55]. OATP1B1 and OATP1B3 mediate the liver uptake of many clinically important drugs and play an important role in DDIs [56]. OATP1B1 and OATP1B3 inhibitors, when administered in combination with lipid-lowering statins, can lead to adverse reactions [57], including severe rhabdomyolysis [58]. The combination of OATP inhibitors can increase plasma exposure to statins and affect pharmacokinetics [59,60].

### 2.3. Organic Cation Transporter (OCT)

OCT is primarily distributed in the liver and kidney, in which OCT1 [61]/OCT3 [62] are enriched in hepatocytes, and OCT2 [63] is preferentially expressed in renal tubules. These transporters mediate the transport of critical endogenous substances and drugs. OCT1, for example, facilitates the hepatic uptake of organic cations, although studies have shown it can also transport neutral compounds or even anionic substrates. Organic cations are primary substrates for OCT1 [64], which mediates their active transport across the hepatocyte membrane. OCT1 is critical for the hepatic uptake of quinolones [61], a class of antibiotics widely studied for their interaction with this transporter. Specifically, quinolones including ciprofloxacin, fleroxacin, gatifloxacin, levofloxacin, moxifloxacin, norfloxacin, ofloxacin, pefloxacin, prulifloxacin, and sparfloxacin have been shown to inhibit hOCT1 [65]. Mechanistically, these drugs act as dual-functional ligands for OCT1; they serve as substrates for cellular uptake at therapeutic concentrations but behave as competitive inhibitors at supra-clinical doses [65].

Although negatively charged levofloxacin has not been identified as an OCT1 substrate, most fluoroquinolones exhibit potent inhibitory effects on OCT1 [65], suggesting that they may act as substrates of this transporter. The net uptake of negatively charged levofloxacin by OCT1 suggested limited OCT1-mediated transport. Based on its concentration-dependent kinetics and low uptake ratio [64], levofloxacin is classified as a poor OCT1 substrate. In contrast, the antimicrobial trimethoprim is actively transported via OCT1; however, its uptake in OCT2 and OCT3 is minimal compared to OCT1 [64]. Nemonoxacin can serve as a substrate for OCT2, with no significant DDIs reported [38]. Previous experiments demonstrated cellular uptake of trimethoprim [66]. In rodents, studies further revealed that the aminoglycoside gentamicin drug induced kidney injury via OCT2-dependent mechanisms [13]. When inhibitory drugs are co-expressed with OCT-transported drugs, they may alter the pharmacokinetics [67]. Research has demonstrated that OCT2-mediated cimetidine transport is inhibited by ranitidine [68]; OCT2-mediated metformin transport is inhibited by sodium channel blockers [69], β-adrenergic receptor antagonists [70], and cimetidine [71]. The oral antidiabetics repaglinide and rosiglitazone inhibit OCT1-mediated metformin transport [72]. Clinical studies indicate that OCT-related DDIs predominantly affect cationic drugs eliminated via renal secretion [73,74]. For example, co-administration of lamivudine with trimethoprim reduces its renal clearance, whereas combining imatinib with cisplatin mitigates cisplatin-induced nephrotoxicity and ototoxicity [75,76,77]. The DDI mediated by these may lead to abnormal elevation of blood drug concentration (for example, trimethoprim inhibiting OCT2/MATE1 can double the blood concentration of digoxin), increasing the risk of poisoning. However, traditional DDI studies are often limited by the inability of in vitro models to accurately simulate the complex physiological environment in vivo. The study [78] proposes that endogenous substrates creatinine and N^1^-methyl nicotinamide (NMN) can be used as biomarkers for OCT2/MATE1 and can be quantified through the physiological pharmacokinetic (PBPK) model to study the interactions mediated by transporters, providing a new strategy for clinical risk assessment.

### 2.4. Oligopeptide Transporter (PEPT)

PEPT1 and PEPT2 are expressed in the brush border membrane of the small intestine and the renal tubule epithelial cells to regulate oligopeptide absorption. These peptide transporters exhibit unique broad substrate specificity and are responsible for the membrane transport of many drugs with distinct chemical structures and medical indications, such as β-lactam antibiotics, cephalosporins, and angiotensin-converting enzyme inhibitors [79,80,81].

PEPT1 is highly abundant in the apical membrane of human intestinal cells throughout the small intestine (absent in the colon) and functions as a low-affinity high-capacity transporter for peptide-like drugs, including β-lactam antibiotics such as amoxicillin [82]. This transporter has been leveraged in innovative drug delivery strategies, where the oral bioavailability of certain drugs is enhanced by administering prodrugs designed as PEPT1 substrates (e.g., cefuroxime axetil) [81]. PEPT2 plays a critical role in the pharmacokinetic disposition of di/tripeptides, as well as peptide-like drugs, in the kidney and brain. Specifically, PEPT2 facilitates the renal reabsorption of cephalexin, cefotaxime, cefaclor, and cephaloridine [83,84,85]. However, unlike PEPT1, limited data exist on species-specific differences in the transport of substrates mediated by PEPT2 [86].

DDIs caused by changes in pharmacokinetics and/or pharmacodynamics may lead to drug-induced toxicity or alter the therapeutic effects of a drug [87]. For example, studies have shown that when betahistine is combined with cefixime, the blood concentration and bioavailability of both are reduced, and the two can competitively inhibit PEPT [88]. Cefaclor is actively secreted via renal PEPT2 for excretion. Probenecid (a uricosuric agent) inhibits PEPT2 function, thereby reducing the excretion of cefaclor and prolonging its half-life [89]. Captopril as a substrate of PEPT1 may share the transport pathway with other substrates (such as cephalexin) when used in combination, resulting in changes in the absorption kinetics [90]. Valacyclovir is absorbed via PEPT1. When used in combination with β-lactam antibiotics (such as amoxicillin), they may compete with the transporter for absorption sites, resulting in reduced absorption of both drugs [91].

**Table 1:** Uptake transporters and antibacterial agents.

Uptake Transporters	Substrates	Inhibitors
OAT1	Cefazolin [33], cefotiam [33,34], cephalexin [33,34], meropenem [37], nemonoxacin [38]	Tetracycline [33], oxytetracycline [33], minocycline [33], doxycycline [33], meropenem–vaborbactam [37]
OAT2	Cefotaxime [36], erythromycin [36], tetracycline [36]	Tetracycline [33], oxytetracycline [33], minocycline [33]
OAT3	Cefdinir [35], cefotiam [35], meropenem [37], nemonoxacin [38], penicillin [40]	Meropenem–vaborbactam [37]
OAT4		Tetracycline [33]
OATP1A1	Nafcillin [54]	
OATP1A2	Ciprofloxacin [47], levofloxacin [47]	
OATP1A4	Nafcillin [54,55], cefradine [54], cefazolin [54], cefmetazole [54], cefoperazone [54], cefsulodin [54]	
OATP1A5	Ciprofloxacin [48]	Azithromycin [51], clarithromycin [51], clindamycin [51]
OATP1B1	Erythromycin [51], clarithromycin [51]	Rifampicin [49], macrolides (except for azithromycin) [52]
OATP1B3	Clarithromycin [51], erythromycin [51]	Rifampicin [50], bilirubin [52], macrolides (except for azithromycin) [52]
OCT1	Trimethoprim [64], ciprofloxacin [65], fleroxacin [65], gatifloxacin [65], levofloxacin [64,65], moxifloxacin [65], norfloxacin [65], ofloxacin [65], pefloxacin [65], prulifloxacin [65], sparfloxacin [65]	Ciprofloxacin [65], fleroxacin [65], gatifloxacin [65], levofloxacin [65], moxifloxacin [3,65], norfloxacin [65], ofloxacin [65], pefloxacin [65], prulifloxacin [65], sparfloxacin [65]
OCT2	Nemonoxacin [38], trimethoprim [64], gentamicin [13,64]	
OCT3		Moxifloxacin [3]
PEPT1	Cefuroxime axetil [81], amoxicillin [82], cefixime [88], cephalexin [83,90], amoxicillin [82,91]	Cefixime [88]
PEPT2	Cephalexin [83,85], cefotaxime [83,85], cefaclor [83,84,85,89], cephaloridine [83,85], cefixime [88]	Cefixime [88]

## 3. Efflux Transporters with Antibacterial Agents

### 3.1. P-Glycoprotein (P-Gp)

P-gp is a multidrug resistance protein that has been extensively studied. It actively transports drugs out of cells and is widely distributed in the body, including the intestine, liver, kidney, brain and placenta [92]. Research has shown that P-gp is a transmembrane protein composed of two symmetrical “half molecules” [93]. Each half molecule contains six transmembrane domains (TMD) and one nucleotide binding domain (NBD). Quinolone antibiotics have a typical 4-quinolone parent nucleus structure, usually with piperazine rings, fluorine atoms, and other substituents, which gives them good antibacterial activity and lipophilicity and easily interacts with the hydrophobic binding pocket of P-gp. After P-gp binds to quinolone drugs, it may promote the drug’s transport out of the cell through the dimerization of NBD and the conformational adjustment of TMD [93]. P-gp-mediated DDIs are frequently observed in clinical settings. For instance, digoxin is a substrate of P-gp, and modulation of P-gp activity (via inhibition or induction) alters its pharmacokinetics. Wakasugi et al. [94] observed an increase in the blood concentrations of digoxin when it was co-administered with clarithromycin. Since P-gp is the sole efflux transporter involved in the renal excretion of digoxin, this suggests that clarithromycin, as a P-gp inhibitor, reduces the renal excretion of digoxin, thereby elevating its systemic exposure. Rengelshausen et al. [95] observed that co-administration of digoxin with clarithromycin increased the oral bioavailability of digoxin but reduced its renal excretion. This suggests that clarithromycin inhibits P-gp-mediated excretion of digoxin in both the intestine and kidneys. Eberl et al. [96] demonstrated in vitro that macrolide drugs (e.g., telithromycin, clarithromycin, roxithromycin, azithromycin, and erythromycin ethyl succinate) inhibit P-gp-mediated efflux transport of digoxin, thereby increasing its systemic concentration. Therefore, macrolides are hypothesized to act as potent P-gp inhibitors, and P-gp-mediated DDIs warrant careful monitoring during clinical use. Aminoglycosides are primarily eliminated via renal excretion, with only a minor fraction undergoing biliary elimination. Consequently, efflux transporters significantly influence their excretion kinetics. However, only one study has demonstrated that P-gp directly mediates tobramycin efflux [97]. The role of efflux transporters in the pharmacokinetics of aminoglycosides requires further investigation. Studies demonstrate that minocycline and riluzole are substrates of P-gp, with minocycline additionally acting as a P-gp inhibitor. Co-administration of minocycline and riluzole was shown to elevate riluzole concentrations in the brain [98]. In Caco-2 cell models, oxytetracycline was identified as both a P-gp substrate and inhibitor of P-gp-mediated efflux for classical substrates such as rhodamine 123 and ivermectin [99]. These findings highlight the need to monitor P-gp-mediated DDIs when tetracyclines are combined with other P-gp substrates. Clinical studies have shown that when apixaban and dabigatran etexilate are used in combination with inhibitors or inducers of P-gp, the pharmacokinetic parameters will undergo significant changes, which can affect the clinical treatment outcome and even increase adverse reactions [43].

### 3.2. Breast Cancer Resistance Protein (BCRP)

BCRP is widely distributed in tissues such as the small intestine, kidney, liver, breast, pancreas, placenta, and blood–brain barrier, playing a critical role in drug pharmacokinetics [18]. It transports a broad spectrum of substrates, including anticancer agents, dietary compounds, and antibiotics, with its substrate specificity partially overlapping that of P-gp and MRP2 [18]. In both rat and human small intestines, BCRP primarily mediates the efflux of ciprofloxacin into the intestinal lumen [100]. In another study using human BCRP-MDCK and mouse Bcrp-MDCK cell models, ciprofloxacin, ofloxacin, and norfloxacin were identified as substrates of these transporters [101]. Pharmacokinetic analysis revealed that the blood concentration of ciprofloxacin in Bcrp1-knockout mice 15min after oral administration was twice as high as in the wild type (1.77 ± 0.73 versus 0.85 ± 0.39 µg/mL, *p* < 0.01). To investigate Bcrp-1-mediated secretion of quinolones into milk, ciprofloxacin (10 mg/Kg) was intravenously administered to lactating female mice (both Bcrp1-knockout and wild-type). The milk was collected and analyzed 10 min post-administration. The results demonstrated that the concentration of ciprofloxacin in milk from Bcrp1-knockout mice was half that of wild-type mice (2.19 ± 0.13 versus 4.44 ± 0.84 µg/mL, *p* < 0.01). This confirms that Bcrp1 mediates the secretion of ciprofloxacin into milk [102]. Interestingly, the researchers also studied the bile excretion of quinolones in Bcrp1-knockout mice and wild-type mice. The results showed that the bile excretion of quinolones such as ciprofloxacin, grepafloxacin, ofloxacin, and prulifloxacin was reduced in Bcrp1-knockout mice to 86%, 50%, 40%, and 16% of the wild type, respectively [103]. Therefore, Bcrp1 plays an important role in the biliary excretion of quinolones. At the same time, Bcrp also mediates the efflux of cefoperazone, cefamandole, ceftriaxone, cefotiam, and other cephalosporins [104]. Febuxostat is an inhibitor of BRCP, and when combined with methotrexate, it improves its pharmacokinetics by increasing systemic exposure to the antifolate [105].

### 3.3. Multidrug Resistance-Associated Protein (MRP)

MRP2 is mainly distributed in the bile duct, renal tubules, and intestinal epithelial cells; MRP3 is mainly distributed on the sinusoidal side of the hepatic blood sinus and is involved in bile excretion. MRP2 substrates are widely excreted, including bound organic anionic compounds such as endogenous glucuronic acid conjugates, steroid hormone glucuronic acid and sulfuric acid conjugates, glutathione conjugates, and phase II metabolites of xenobiotics [106]. MRP2 is involved in the efflux process of β-lactam drugs. For example, one study using vesicles expressing rMrp2 and hMRP2 showed that cefoperazone, cefpiramide, and ceftriaxone exhibited significantly greater efflux than empty vesicles. In vesicles transfected only with hMRP2, the efflux of cefotetan and cefotiam was also significantly increased compared to empty vesicles [102]. The above studies have shown that cephalosporins are substrates of Mrp and MRP. Kwatra D et al. found that erythromycin intake in MDR1-MDCKII and MRP2-MDCK cells was significantly increased during gatifloxacin co-administration, resulting in 1.6- and 1.7-times higher accumulation compared to single-agent erythromycin [102]. This suggests that gatifloxacin, as a potent P-gp and MRP2 inhibitor, potentially modulates the pharmacokinetics of co-administered drugs. Concurrently, another experiment also confirmed that gemifloxacin is a substrate for P-gp and MRP2. Gemifloxacin inhibited erythromycin efflux from P-gp and MRP2 in a concentration-dependent manner, with IC50 values of 123 ± 2 µM and 16 ± 2 µM, respectively [107]. In Caco-2 cells, co-treatment with P-gp inhibitors PSC833, GF120918, and the MRP inhibitor MK571 reduced the secretion of danofloxacin mesylate while increasing its absorption rate. This demonstrates that danofloxacin mesylate is a substrate of both P-gp and MRP2 [108].

### 3.4. Mammal Multidrug and Toxin Extrusion Protein (MATE)

MATEs are mainly expressed in the bile duct surface of hepatocytes and the brush border membrane of renal tubules, which pump substrates out of cells and reduce their concentration in the liver and kidneys [109,110]. MATE transporters catalyze the active expulsion of a variety of chemically and structurally distinct compounds, including antimicrobial agents and chemotherapy drugs, thereby promoting multidrug resistance in pathogenic bacteria and cancers [109]. Research on MATEs remains in its early stage yet warrants significant attention. MATE1 is primarily expressed in the liver and kidneys, whereas MATE2 and MATE2-K are mainly expressed in the kidneys—MATE2-K uniquely and MATE2 additionally in the placenta [111].

MATE1 is a multi-specific transporter that exhibits overlapping specificity with OCT2 or other OCTs as cationic substrates [111]. In the kidneys, metformin is taken up by OCT2 into renal cells and excreted into urine by MATE1/2K; approximately 50% of the parent drug is eliminated unchanged. Moxifloxacin significantly inhibited metformin uptake in OCT1, OCT3, MATE1, and MATE2-K transfected cells, reducing accumulation to 52%, 39%, 12%, and 16% of single-treatment levels, respectively [3]. In particular, moxifloxacin inhibited the uptake of metformin by more than 70%, with IC50 values of 12 µM and 7.6 µM in MATE1 and MATE2-K cells, respectively [3]. Therefore, moxifloxacin is a potent inhibitor of MATE1 and MATE2-K. The expression of MATE transporters in pathogens confers resistance to fluoroquinolones (norfloxacin, ciprofloxacin), aminoglycosides (kanamycin, erythromycin), and even the new generation of glycylcycline antibiotics [109]. The Ki value of metformin uptake via OCT2 and MATE1 inhibited by trimethoprim was more than 50% higher than the estimated peak plasma concentration (C_max_) of unbound trimethoprim in systemic blood. The Ki value of MATE2-K-mediated metformin uptake was reduced by 63%, indicating trimethoprim’s inhibitory effect on MATE2-K. Therefore, changes in the renal secretion of metformin after trimethoprim administration may be related to the inhibition of apical MATEs rather than basolateral OCT2 [111]. Clinical studies have also shown that famotidine, by inhibiting MATE1, can enhance the hypoglycemic effect of metformin [43].

**Table 2:** Efflux transporters and antibacterial agents.

Efflux Transporters	Substrates	Inhibitors
P-gp	Tobramycin [98], minocycline [98], oxytetracycline [99], ivermectin [99], gemifloxacin [107]	Clarithromycin [94,96], telithromycin [96], roxithromycin [96], azithromycin [96], erythromycin [96], minocycline [98], oxytetracycline [99], gatifloxacin [102], gemifloxacin [107]
BCRP	Ciprofloxacin [100,101,103], ofloxacin [101,103], norfloxacin [101], grepafloxacin [103], prulifloxacin [103], cefoperazone [104], cefamandole [104], ceftriaxone [104], cefotiam [104]	
MRP2	Cefoperazone [102], cefpiramide [102], ceftriaxone [102], cefotetan [102], cefotiam [102], erythromycin [102], gemifloxacin [107], danofloxacin mesylate [108]	Gatifloxacin [102], gemifloxacin [107]
MATE1	Norfloxacin [109], ciprofloxacin [109], kanamycin [109], erythromycin [109]	Moxifloxacin [3]
MATE2-K	Norfloxacin [109], ciprofloxacin [109], kanamycin [109], erythromycin [109]	Moxifloxacin [3]

## 4. Cytochrome P450 Enzymes with Antibacterial Agents

**Figure 2:**
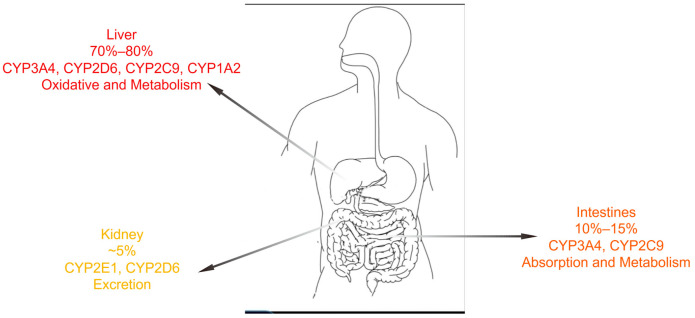
Major CYP enzymes in the body.

Most drugs undergo CYP450-mediated metabolism in the intestine or liver [112]. The inhibition or induction of the CYP enzyme system can alter the metabolism of other drugs and is a major mechanism underlying drug interactions. At least seven subtypes of CYP enzymes (CYP1A2, CYP2C9, CYP2C19, CYP2D6, CYP2E1, and CYP3A4/5) mediate the oxidation of a wide range of drugs, including lipid-lowering agents, cardiovascular drugs, and antibacterial agents [113]. The distribution of CYP450 in the body is illustrated in Figure 2. Common inhibitors such as erythromycin, ciprofloxacin, enoxacin, fluconazole, itraconazole, and voriconazole should be used cautiously when co-administered with other medications, particularly those with a narrow therapeutic index such as warfarin, cyclosporine, tacrolimus, and isoniazid [113,114]. CYP1A2 has a relatively small substrate-binding site, and its substrates and inhibitors are typically small, lipophilic, and planar molecules, such as ciprofloxacin, a potent inhibitor [115]. Warfarin is a prototypical substrate for CYP2C9, an enzyme responsible for metabolizing over 100 drugs and endogenous compounds. Concomitant use of fluconazole, a potent CYP2C9 inhibitor, significantly inhibits warfarin metabolism and increases the risk of bleeding [116]. Omeprazole, a CYP2C19 inhibitor, interferes with the bioactivation of clopidogrel, reducing the area under the concentration–time curve (AUC) of clopidogrel’s active metabolite [117]. Quinidine is a specific and potent inhibitor of CYP2D6 [118]. Clinically important drugs, including anticancer agents (e.g., tamoxifen and irinotecan), antibacterials (e.g., clarithromycin, erythromycin and isoniazid), antihypertensives (e.g., dihydralazine, verapamil, and diltiazem), sex steroids and their receptor modulators (e.g., gestodene and raloxifene), anti-HIV agents (e.g., ritonavir and delavirdine), and several herbal components (e.g., bergamottin and glabridin), exhibited a distinct inhibitory effect on CYP3A4 [119].

Inhibition or induction of CYP450 enzyme activity can lead to DDIs [120]. More than 75% of drug metabolism is mediated by the CYP superfamily. CYP3A, the most abundant CYP isoform in humans, plays a pivotal role in drug metabolism [121,122]. Most drugs are either inactivated through metabolic processes or excreted following biotransformation. When a drug acts as a CYP inducer, it accelerates the metabolism of co-administered drugs that share the same CYP isoenzyme, leading to their rapid excretion from the body. Consequently, accelerated clearance may reduce plasma drug concentrations below the therapeutic threshold, resulting in treatment failure. Conversely, CYP inhibitors may cause co-administered drugs to accumulate to toxic concentrations, increasing the risk of overdose and adverse effects [120,123]. Clarithromycin, a potent CYP3A4 inhibitor, significantly increases the area under the AUC of simvastatin and lovastatin when co-administered, as demonstrated in clinical studies [124]. Rifampicin, a CYP3A4-mediated metabolism of cyclosporine, resulted in subtherapeutic drug levels [125]. Co-administration of fluconazole with fluvastatin may increase fluvastatin exposure (elevated AUC and C_max_). Although no severe adverse effects have been reported, cautious use is recommended to avoid potential toxicity [126].

**Table 3:** CYP450 enzymes and antibacterial agents.

CYP450 Enzymes	Substrates	Inhibitors
CYP1A2		Ciprofloxacin [115]
CYP2C9		Fluconazole [34,114,116,126]
CYP2C19		Fluconazole [34,114,116,126],
CYP3A4	Erythromycin [114], rifampicin [125], fluconazole [126]	Erythromycin [114], ciprofloxacin [114], enoxacin [114], fluconazole [34,114,116,126], itraconazole [114], voriconazole [114], clarithromycin [119,124], erythromycin [119], isoniazid [119], ritonavir [119], delavirdine [119]

## 5. Conclusions

In summary, drug transporters and metabolic enzymes exert critical roles in modulating the pharmacokinetic behavior of antimicrobial agents, profoundly influencing their therapeutic efficacy and safety profiles. Given the prevalent clinical practice of co-prescribing antimicrobials with concomitant medications, the meticulous evaluation of transporter- and enzyme-mediated DDIs is imperative. Competitive inhibition at shared molecular targets (e.g., CYP3A4, P-gp) can perturb the pharmacokinetic and pharmacodynamic profiles of co-administered drugs, potentiating the risks of hepatotoxicity, nephrotoxicity, or therapeutic failure. These findings provide a critical foundation for optimizing combination therapies and advancing rational drug use in clinical settings.

## Data Availability

Not applicable.
